# The feasibility and effects of a pharmacological treatment algorithm for cancer patients with terminal dyspnea: A multicenter cohort study

**DOI:** 10.1002/cam4.5362

**Published:** 2022-10-19

**Authors:** Masanori Mori, Takashi Yamaguchi, Kozue Suzuki, Yoshinobu Matsuda, Ryo Matsunuma, Hiroaki Watanabe, Tomoo Ikari, Yoshihisa Matsumoto, Kengo Imai, Naosuke Yokomichi, Satoru Miwa, Toshihiro Yamauchi, Soichiro Okamoto, Satoshi Inoue, Akira Inoue, Tatsuya Morita, Eriko Satomi, the Japanese Dyspnea Relief Investigators

**Affiliations:** ^1^ Seirei Mikatahara General Hospital Hamamatsu Japan; ^2^ Kobe University Graduate School of Medicine Kobe Japan; ^3^ Tokyo Metropolitan Komagome Hospital Bunkyo‐ku Japan; ^4^ National Hospital Organization Kinki‐Chuo Chest Medical Center Sakai Japan; ^5^ Konan Medical Center Kobe Japan; ^6^ Komaki City Hospital Komaki Japan; ^7^ Tohoku University School of Medicine Sendai Japan; ^8^ Cancer Institute Hospital of Japanese Foundation for Cancer Research Tokyo Japan; ^9^ Seirei Hospice, Seirei Mikatahara General Hospital Hamamatsu Japan; ^10^ National Cancer Center Hospital Tokyo Japan

**Keywords:** algorithm, cancer, dyspnea, feasibility, palliative care

## Abstract

**Background:**

How clinicians treat patients with terminal dyspnea widely varies, which could hamper quality care. We visualized comprehensive pharmacological treatment delivered by palliative care physicians.

**Aim:**

To examine adherence to a comprehensive pharmacological treatment algorithm for patients with terminal dyspnea, and to explore its outcomes during 48 h.

**Design:**

A multicenter cohort study at five sites (February 2020 to June 2021).

**Setting/Participants:**

We prospectively enrolled consecutive patients with advanced cancer, Eastern Cooperative Oncology Group performance status 3–4, and moderate/severe dyspnea. Participating palliative care physicians initiated algorithm‐based treatment. The primary outcome was the proportion of adherence to the treatment algorithm over 24 h (predefined goal, 70%). We evaluated the adherence, goal achievement, and dyspnea level with a numerical rating scale (NRS), as well as adverse events over 48 h.

**Results:**

All 108 patients received algorithm‐based pharmacological treatment. Among 96 and 87 patients who were alive at 24 and 48 h, respectively, 96 (100%; 95% confidence interval [CI] = 96%–100%) and 82 (94%; 95%CI = 87%–98%) continued to receive the algorithm treatment, respectively, and 66 (69%; 95%CI = 59%–77%) and 64 (74%; 95%CI = 63%–82%) achieved the treatment goals, respectively. Using a complete case analysis with paired t‐tests, mean dyspnea NRS scores significantly reduced from 7.3 (standard error, 0.2) at the baseline to 4.9 (0.3) at 24 h (*n* = 72; *p* < 0.001), and 7.2 (0.3) at the baseline to 4.6 (0.4) at 48 h (*n* = 55; *p* < 0.001). Most adverse events were mild to moderate.

**Conclusions:**

The comprehensive pharmacological treatment algorithm was feasible, and the study data supports its preliminary efficacy and safety. The use of this algorithm may help clinicians improve care for patients with terminal dyspnea.

## Graphical abstract

We visualized a comprehensive pharmacological treatment algorithm that includes various treatment options for cancer patients with terminal dyspnea. This multicenter cohort study showed that the treatment algorithm was highly feasible, and correlated with a significant improvement in dyspnea with rare adverse events.

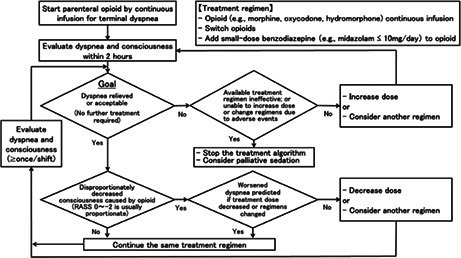

## 1 INTRODUCTION

Dyspnea is among the most frequent and deteriorating symptoms in the terminal phase of life of cancer patients (“terminal dyspnea”).^1^, ^2^, ^3^ Previous research indicated the efficacy of opioids as the first‐line therapy for terminal dyspnea patients.^4^, ^5^, ^6^, ^7^, ^8^ The international guidelines also recommend that parenteral opioids and benzodiazepines be considered for patients with terminal dyspnea.^9^, ^10^, ^11^ However, little evidence exists regarding how best to use these medications comprehensively.^2^, ^12^, ^13^ Moreover, while mental awareness is considered important in the last phase of life, how best to balance between dyspnea and communication capacity is poorly understood.^14^, ^15^

**FIGURE 1:**
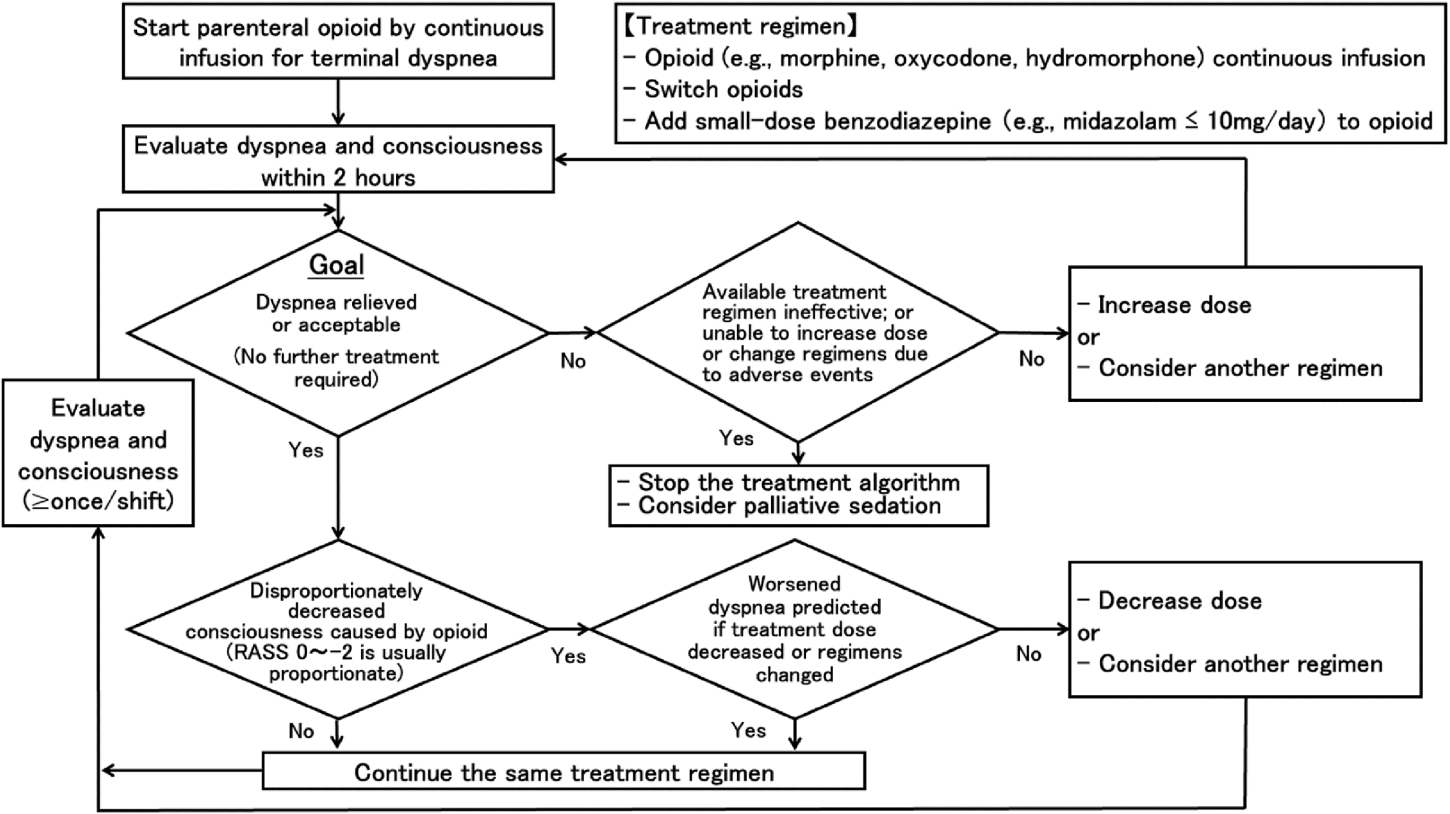
The comprehensive pharmacological treatment algorithm for terminal cancer dyspnea patients. RASS, Richmond Agitation‐Sedation Scale

On bedside, physicians address these complex issues individually, but how they manage terminal dyspnea varies substantially, which could hamper the quality of care.^5^, ^7^, ^16^ An algorithm that visualizes comprehensive pharmacological treatment by palliative care physicians would be useful. Such an algorithm could help minimize the variability and promote optimal care, facilitate training and education, and provide a model of treatment for future research. While our prior study proposed an opioid titration algorithm,^17^ it involved only opioids as treatment options and proxy measurements. In addition, it reported only 24‐h outcomes. In clinical practice, physicians occasionally switch opioids and/or add benzodiazepines as the second‐line treatment after titration of the initial opioid for terminal dyspnea.^16^ Thus, we have expanded the opioid titration algorithm,^17^ included comprehensive pharmacological treatment options (Figure 1)^10^, ^11^, ^16^ and patient‐reported outcomes as well as goal achievement, and herein report longer‐term outcomes.

The primary aim of this study was to examine adherence to the comprehensive pharmacological treatment algorithm for patients with terminal dyspnea. Secondary aims were to examine its efficacy and safety over 48 h. We hypothesized that the treatment algorithm would be feasible, effective, and safe for patients with terminal dyspnea.

## 2 METHODS

### 2.1 Design

We conducted a multicenter prospective cohort study involving advanced cancer patients seen by palliative care physicians in inpatient consultation teams and inpatient hospices/palliative care units at five sites in Japan. All assessment and interventions were conducted within routine practice. The study was conducted based on the ethical standards of the Helsinki Declaration and the ethical guidelines for medical and health research involving human subjects presented by the Ministry of Health, Labour, and Welfare in Japan. We obtained approval by the Institutional Review Boards (IRBs) of the main institution (Seirei Mikatahara General Hospital, No.19–57, January 20, 2020) and all the other study sites. The requirement for written informed consent in this observational study was waived by the IRBs based on the ethical guidelines.

### 2.2 Participants

We enrolled patients consecutively from February 2020 to June 2021. Inclusion criteria included: age ≥ 18 years old; advanced cancer; an Eastern Cooperative Oncology Group (ECOG) performance status (PS) ≥ 3; moderate‐overwhelming dyspnea (the Integrated Palliative care outcome Scale [IPOS] score of 2–4); maintained communication capacity (0–2 based on the Communication Capacity Scale [CCS], item 4); and initiation of parenteral opioids by continuous administration the first time for dyspnea. Patients were excluded if they received treatment for dyspnea caused by etiologies unrelated to cancer, or if they were scheduled to undergo an intervention that would markedly influence dyspnea intensity (e.g., thoracentesis for pleural effusion). The patients were followed for 1 month or until death, whichever came first.

### 2.3 Treatment strategies

We chose morphine, oxycodone, and hydromorphone as frequently used opioids for dyspnea in Japan.^18^ A pharmacological treatment algorithm was developed that visualizes how palliative care specialists use parenteral opioids by continuous administration, based on our preliminary treatment algorithm, review of the literature, and discussions among the researchers, many of whom are active committee members of the national and/or international guidelines for cancer dyspnea (Figure 1).^11^, ^16^, ^17^, ^19^ We did not include titration of oral opioids in the algorithm, as terminally‐ill cancer patients with severe dyspnea often lose consciousness and become unable to take medications orally.^3^, ^5^, ^8^ The algorithm reflects a comprehensive treatment strategy that includes opioid initiation and titration, opioid switching, and the addition of low‐dose benzodiazepine. As our primary intention was to provide a visual flow chart of comprehensive treatment strategies, and the most effective doses may change over time as new evidence emerges, we did not include specific values of medication doses in the algorithm. Instead, we indicated in the study protocol that the recommended initial dose of parenteral opioids was 6–12 mg daily (parenteral morphine‐equivalent dose) for opioid‐naïve patients, and 20%–50% increase of prior opioids for opioid‐tolerant patients based on our preliminary study.^17^ Thereafter, the doses were maintained, increased by 20%, or decreased by 20%, as clinically appropriate.^17^ Whether patients were on a benzodiazepine at baseline, the addition of small‐dose benzodiazepine (e.g., midazolam ≤10 mg/day intravenously or subcutaneously) was considered when patients continued to suffer dyspnea despite titration of parenteral opioids, and/or when further titration of opioids would be considered clinically controversial because uncomfortable side effects developed (e.g., drowsiness, respiratory depression, delirium, myoclonus, nausea and vomiting).^16^ The overall aim of the treatment algorithm was to achieve rapid relief of dyspnea while maintaining the consciousness level or communication capacity.^2^, ^5^, ^8^, ^17^, ^19^ The decision whether to utilize the algorithm for individual patient was made by each physician based on his or her preference. All physicians were allowed to adjust the doses as appropriate (e.g., organ functions, patient and family preferences), and to provide concurrent palliative treatment for dyspnea.

### 2.4 Measurements

We recorded measurement variables at the baseline, 24 h, and 48 h by responsible physicians.

#### 2.4.1 Baseline data

Baseline data of the patients included patients' basic demographics, etiologies of dyspnea, comorbidities, and various signs and symptoms. We also collected laboratory data to evaluate baseline renal and hepatic functions. Other variables including the prior use of opioids and use of the treatment algorithm were obtained.

#### 2.4.2 Adherence to the treatment algorithm

We calculated the percentage of patients who were provided with the algorithm‐based treatment, and percentage of those who continued to receive it over 48 h (i.e., adherence rate). We also documented whether the algorithm was discontinued because of dyspnea relief or adverse events. The treatment algorithm was considered feasible a priori if the adherence rate was greater than 70%. We chose an adherence rate of 70% or greater as our feasibility threshold, as our previous pilot study that explored the feasibility of a visual treatment algorithm of parenteral opioids applied the same cut‐off, and showed that the threshold of 70% could be achieved in cancer patients with terminal dyspnea.^17^

#### 2.4.3 Efficacy

The dyspnea intensity was evaluated with a numerical rating scale (NRS) by patients, and NRS and IPOS scores rated by physicians at the baseline (worst over 4 h) as well as at 24 and 48 h (worst over 2 h at each time‐point).^5^, ^20^, ^21^ Dyspnea NRS is one component of the reliable and validated Edmonton Symptom Assessment System, which is a 11‐point numerical scale, ranging from 0 (no dyspnea) to 10 (worst possible dyspnea).^22^ The minimally clinically important difference (MCID) of NRS is one for cancer dyspnea.^23^, ^24^ IPOS is a reliable and validated proxy scale for symptom assessment.^25^, ^26^ How much a patient was affected by dyspnea was evaluated on a 5‐point scale (0 = not at all, to 4 = overwhelmingly). Physicians also assessed improvement using the Clinical Global Impression scale – improvement (CGI‐I).^27^ CGI‐I is a validated 7‐point scale ranging between 1 (very much improved) and 7 (very much worse).^28^ We adopted these physician‐reported outcomes, as patients' cognitive capacity often deteriorates as death approaches.^2^, ^4^, ^5^ The respiratory rate was also collected.

#### 2.4.4 Goal achievement

Achievement of treatment goals was operationally defined as “the case where dyspnea is alleviated or is acceptable to the patient, and the patient does not wish for further adjustment of treatment for dyspnea” based on clinical experiences. If the patient had difficulty communicating, and his or her family was present, the responsible physician asked the family if the patient looked comfortable and would not wish for further adjustment based on observation. If the patient had difficulty communicating and the family was not present, the responsible physician determined whether the treatment goals shared by the patient/family at the initiation of treatment had been achieved.

#### 2.4.5 Consciousness and communication

We assessed the modified Richmond Agitation‐Sedation Scale (RASS) to examine the consciousness level, and CCS item 4 to assess communication capacity. The modified RASS is a reliable and valid tool that measures the severity of agitation and sedation on a 10‐point scale (−5 = unarousable, to +4 = combative).^29^, ^30^, ^31^, ^32^ CCS is a reliable and valid 5‐item tool to evaluate the communication capacity of advanced cancer patients.^33^ Item 4 of the CCS quantifies voluntary communication on a 4‐point scale ranging from 0 (voluntary and explicit communication of complex contents) to 3 (unable to communicate).^5^, ^15^

#### 2.4.6 Adverse events

The severity of adverse events including nausea, delirium, and apnea was assessed with the Common Terminology Criteria for Adverse Events version 4.0 over 48 h.

### 2.5 Statistical analyses

The primary outcome was the rate of adherence to the treatment algorithm over 24 h. The secondary outcomes included adherence at 48 h as well as efficacy, goal achievement, modified RASS, CCS item 4, and adverse events at 24 and 48 h.

We used descriptive statistics to summarize baseline data and outcomes over time. We then performed paired t‐tests to demonstrate changes over time in patients who remained alive at 24 and 48 h as compared with the baseline. In some patients, NRS scores become unevaluable essentially due to the development of cognitive impairment. Thus, we first analyzed existing data without conducting imputation (complete case analysis). Next, we conducted sensitivity analysis with the missing data replaced by the last observation data of the same patients (last‐observation‐carried‐forward [LOCF] analysis), as complete case analysis could lead to selection bias. Dyspnea relief at 24 and 48 h was defined as dyspnea IPOS score of 0 or 1.^5^, ^8^, ^17^

To assess a balanced outcome, we grouped patients based on the dyspnea intensity and communication capacity as follows^2^, ^5^, ^8^, ^17^: complete relief (no or minimal dyspnea (IPOS = 0–1) and full communication capacity (CCS item 4 = 0)); partial relief (moderate dyspnea (IPOS = 2) and/or impaired communication capacity (CCS item 4 = 1–2)); persistent dyspnea (severe dyspnea [IPOS = 3–4] regardless of communication capacity); and unable to communicate (dyspnea IPOS 0–2 and CCS item 4 = 3). We also evaluated the percentage of patients with each category at 24 and 48 h among patients who had severe baseline dyspnea (IPOS≥3).

We used Kaplan–Meier methods to calculate the median survival time from the day when parenteral opioids were started for dyspnea. Those who remained alive at 1 month were treated as censored.

To achieve an adherence rate ≥ 70%, 81 patients would be required to calculate accuracy within a 20% width at a 95%CI. Considering potential missing data and attrition in patients close to death, we aimed to enroll 100 patients. A *p*‐value<0.05 (two‐sided) was considered significant. The Statistical Package for the Social Sciences, version 25.0 (SPSS Inc., IBM, JAPAN) was used for statistical analyses.

## 3 RESULTS

A total of 108 patients were enrolled (Table 1). The mean age was 72 (standard deviation [SD], 12), 43 (40%) patients had lung cancer, 59 (55%) had lung metastases, and 85 (79%) had an ECOG PS of 4. The median overall survival was 6 days (95%CI = 4.8–7.2).

**TABLE 1:** Baseline characteristics (*n* = 108)

Variables	Total (*n* = 108)
Age, mean (SD)	72 (12)
Sex, female	50 (46%)
Primary tumor	
Lung	43 (40%)
Gastrointestinal tract (esophagus/stomach/colon/rectum)	15 (14%)
Breast	12 (11.1%)
Blood and lymph node	8 (7.4%)
Pancreas and biliary	7 (6.5%)
Ovary and uterine	6 (5.6%)
Kidney, ureter, bladder, and prostate	3 (2.8%)
Other	14 (13%)
Metastatic lesions	
Any	102 (94%)
Pleural effusions	64 (59%)
Lung	59 (55%)
Pleura	55 (51%)
Liver	28 (26%)
Central nervous system	7 (6.5%)
Comorbidity	
Chronic obstructive pulmonary disease	10 (9.3%)
Interstitial lung diseases	7 (6.5%)
Dementia	5 (4.6%)
Congestive heart failure	1 (0.9%)
Cerebrovascular disorders	1 (0.9%)
ECOG PS on administration of parenteral opioid for dyspnea	
3	23 (21%)
4	85 (79%)
Palliative performance scale	
10	12 (11%)
20	41 (38%)
30	25 (23%)
40	22 (20%)
50	7 (6.5%)
Missing	1 (0.9%)
Etiology of dyspnea	
Pulmonary tumors (primary, metastasis)	42 (39%)
Pleural effusions	39 (36%)
Lymphangitis carcinomatosis	19 (18%)
Cachexia/respiratory muscle fatigue	13 (12%)
Respiratory infection	11 (10%)
Difficulty in expectoration	1 (0.9%)
Anemia	1 (0.9%)
Other	18 (17%)
Unknown	2 (1.9%)
Oral intake	
Normal	4 (3.7%)
Decreased	34 (32%)
A few mouthfuls	70 (65%)
Edema, yes	55 (51%)
Delirium, yes	23 (21%)
Concurrent treatment for dyspnea at baseline	
Supplemental oxygen, yes	94 (87%)
Corticosteroids, yes	53 (49%)
Prior use of regular opioid, yes	60 (56%)
Types of prior opioids	
Morphine	17 (28%)
Oxycodone	20 (33%)
Hydromorphone	9 (15%)
Fentanyl	11 (18%)
Other	3 (5.0%)
Prior opioid dose in patients on regular opioids (oral MEDD, mg/day)	(*n* = 60)
Mean (SD)	
Median (IQR)	63 (88) 35 (16, 60)

Abbreviations: ECOG PS, Eastern Cooperative Oncology Group Performance Status; IQR, interquartile range; MEDD, morphine‐equivalent daily dose; SD, standard deviation.

### 3.1 Adherence rate to and contents of the treatment algorithm

All 108 patients (100%) received the algorithm‐based treatment (Table 2). Among 96 and 87 patients who were alive at 24 and 48 h, respectively, 96 (100%; 95%CI = 96%–100%) and 82 (94%; 95%CI = 87–98%) adhered to the algorithm, respectively. At 48 h, 5 patients discontinued the treatment algorithm due to the development of adverse events and requirement of palliative sedation.

**TABLE 2:** Actual treatment over 48 h

	Baseline (*n* = 108)	24 h (*n* = 96)	48 h (*n* = 87)
Algorithm utilized			
Yes	108 (100%)	96 (100%)	82 (94%)
No	0	0	5 AE (drowsiness), *n* = 1 Palliative sedation, *n* = 4
Opioid			
Morphine	66 (61%)	60 (63%)	54 (63%)
Oxycodone	34 (32%)	31 (32%)	27 (31%)
Hydromorphone	8 (7.4%)	5 (5.2%)	5 (5.8%)
MEDD iv/sc mg/day^a^			
Naïve			
Mean (SD)	7.4 (2.6)	9.4 (4.9)	11 (5.0)
Median (IQR)	6 (6, 7.4)	7.2 (6, 12)	12 (6, 12)
Tolerant			
Mean (SD)	30 (37)	31 (33)	36 (34)
Median (IQR)	18 (12, 30)	18 (12, 36)	24 (18, 38)
Opioid switching	—	0	0
Benzodiazepine	4 (3.7%)	5 (5.2%)	9 (10%)
Alprazolam	1	1	1
Etizolam	1	1	0
Midazolam	0	2	7
Flunitrazepam	2	1	1

Abbreviations: AE, adverse events; MEDD, morphine‐equivalent daily dose; iv, intravenous; sc, subcutaneous; SD, standard deviation; IQR, interquartile range.

^a^ Parenteral morphine and oxycodone doses are equianalgesic.

As parenteral opioids for dyspnea, morphine was used in 66 (61%) patients, followed by oxycodone and hydromorphone in 34 (32%) and 8 (7.4%) patients, respectively (Table 2). Overall, the doses of opioids were increased over 48 h. Opioid switching for dyspnea was not performed over 48 h. The proportion of patients who received benzodiazepines increased over time.

### 3.2 Changes in outcomes

#### 3.2.1 Dyspnea intensity

Table 3 shows the changes in NRS scores of dyspnea after the algorithm‐based treatment was started, with the unevaluable NRS data at 24 and 48 h excluded. The mean patient‐rated dyspnea NRS scores significantly reduced from 7.3 (standard error [SE], 0.2) at the baseline to 4.9 (0.3) at 24 h (*n* = 72; *p* < 0.001), and 7.2 (0.3) at the baseline to 4.6 (0.4) at 48 h (*n* = 55; *p* < 0.001). The results were similar when the unevaluable data of patient‐rated NRS scores at 24 and 48 h were replaced with the last observation data. Similar trends were noted when dyspnea intensity was evaluated by physician‐rated NRS and IPOS (Table 3).

**TABLE 3:** Changes in outcomes from baseline among patients who were alive at 24 and 48 h

		Baseline	24 h	Difference	*p*‐value^a^		Baseline	48 h	Difference	*p*‐value^a^
	*n*	Mean (SE)	Mean (SE)	Mean (95% CI)		*n*	Mean (SE)	Mean (SE)	Mean (95% CI)	
Dyspnea NRS (patient‐rated)										
CC analysis^b^	72	7.3(0.2)	4.9 (0.3)	2.4 (1.8, 3.0)	<0.001	55	7.2 (0.3)	4.6 (0.4)	2.5 (1.9, 3.2)	<0.001
LOCF analysis	82	7.2(0.2)	5.1 (0.3)	2.1 (1.6, 2.6)	<0.001	75	7.2 (0.2)	4.8 (0.3)	2.5 (1.9, 3.1)	<0.001
Dyspnea NRS (physician‐rated)										
CC analysis^b^	94	6.7 (0.2)	4.2 (0.3)	2.5 (2.1, 3.0)	<0.001	82	6.7 (0.2)	3.5 (0.3)	3.1 (2.7, 3.6)	<0.001
LOCF analysis	95	6.7 (0.2)	4.2 (0.3)	2.5 (2.1, 3.0)	<0.001	86	6.6 (0.2)	3.5 (0.3)	3.1 (2.6, 3.6)	<0.001
Dyspnea IPOS	96	3.0 (0.1)	1.7 (0.1)	1.3 (1.1, 1.5)	<0.001	87	2.9 (0.1)	1.4 (0.1)	1.5 (1.3, 1.7)	<0.001
CCS, item 4	96	0.5 (0.1)	0.8 (0.1)	−0.3 (−0.4, − 0.1)	0.002	87	0.5 (0.1)	1.0 (0.1)	−0.5 (−0.7, −0.3)	<0.001
RASS	96	−0.1 (0.1)	−0.4 (0.1)	0.3 (0.1, 0.6)	0.006	87	−0.1 (0.1)	−0.7 (0.2)	0.7 (0.4, 1.0)	<0.001

Abbreviations: SE, standard error; CI, confidence interval; CC, complete case; LOCF, last observation carried forward; IPOS, Integrated Palliative care Outcome Scale; CCS, Communication Capacity Scale; RASS, Richmond Agitation‐Sedation Scale.

^a^ Both *p*‐values were calculated based on comparison with the baseline data.

^b^ Missing data at 24 and 48 h were excluded.

#### 3.2.2 Consciousness and communication

Of patients who were alive at 24 and 48 h, RASS scores significantly reduced from baseline (*p* = 0.006 at 24 h and *p* < 0.001 at 48 h) and CCS item 4 scores significantly increased over time (*p* = 0.002 at 24 h and *p* < 0.001 at 48 h) (Table 3).

Table 4 shows the distribution of various efficacy and safety outcomes over 48 h. Overall, preliminary efficacy was indicated by high percentage of goal achievement, improved patient‐ and physician‐rated dyspnea intensities, and decreased respiratory rate. Moreover, most adverse events were tolerable.

**TABLE 4:** Distribution of outcomes over 48 h

	Baseline (*n* = 108)	24 h (*n* = 96)	48 h (*n* = 87)
Goal achieved, *n* (%; 95% CI)		66 (69%; 58%–77%)	64 (74%; 63%–82%)
NRS (patient‐rated), mean	(*n* = 91)	(*n* = 74)	(*n* = 56)
	7.3 (2.1)	5.0 (2.6)	4.6 (2.7)
NRS (physician‐rated), mean	(*n* = 107)	(*n* = 94)	(*n* = 82)
	6.9 (1.8)	4.2 (2.5)	3.5 (2.3)
IPOS (worst)			
0	0	4 (4.2%)	5 (5.7%)
1	0	45 (47%)	51 (59%)
2	23 (21%)	27 (28%)	21 (24%)
3	63 (58%)	15 (16%)	9 (10%)
4	22 (20%)	5 (5.2%)	1 (1.1%)
IPOS, mean	3.0 (0.6)	1.7 (1.0)	1.4 (0.8)
Physician‐rated improvement			
Much improved		9 (9.4%)	10 (12%)
Improved		45 (47%)	44 (52%)
Sightly improved		24 (25%)	20 (23%)
Unchanged		14 (15%)	8 (9.3%)
Slightly worsened		1 (1.0%)	2 (2.3%)
Worsened		2 (2.1%)	0
Much worsened		1 (1.0%)	2 (2.3%)
Respiratory rate (SD)	22 (7.0)	17 (5.9)	16 (5.3)
Adverse events (CTCAE)			
Nausea			
1	8 (7.4%)	6 (6.3%)	3 (3.4%)
2	7 (6.5%)	0	1 (1.1%)
3	2 (1.9%)	0	0
Delirium			
1	13 (12%)	9 (9.4%)	12 (14%)
2	8 (7.4%)	9 (9.4%)	9 (10%)
3	3 (2.8%)	1 (1.0%)	1 (1.1%)
Apnea			
3	0	1 (1.0%)	1 (1.1%)

Abbreviations: CI, confidence interval; NRS, Numerical Rating Scale; IPOS, Integrated Palliative care Outcome Scale; CTCAE, Common Terminology Criteria for Adverse Events, version 5.0.

#### 3.2.3 Goal achievement

Among 96 and 87 participants who were alive at 24 and 48 h, respectively, 66 (69%; 95%CI = 59–77%) and 64 (74%; 95%CI = 63%–82%) achieved goals of dyspnea relief, respectively (Table 4).

#### 3.2.4 Distribution of other dyspnea outcomes

Among 96 and 87 patients who survived 24 and 48 h, respectively, dyspnea relief was achieved (IPOS≤1) in 49 (51%) and 56 (64%) patients, respectively (Table 4). Physicians rated dyspnea was improved or much improved in 54 (56%) and 54 (62%) of patients at 24 and 48 h, respectively. Respiratory rates also decreased over time.

**FIGURE 2:**
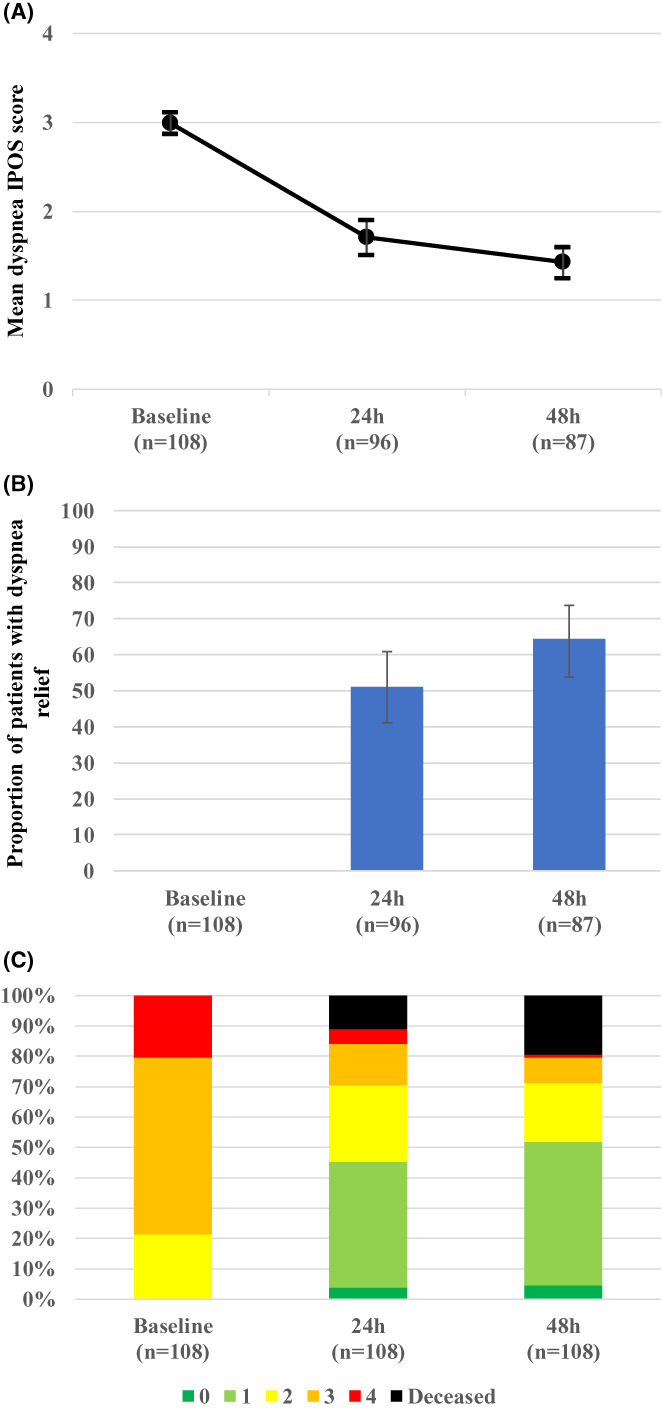
The changes in dyspnea intensities over 48 (A) Changes in mean dyspnea IPOS scores. Bars show 95% confidence intervals. (B) Proportion of patients with dyspnea relief. Dyspnea relief is defined as dyspnea IPOS scores of 0 or 1. Bars show 95% confidence intervals. (C) Distribution of dyspnea IPOS scores in all patients. The number shows dyspnea IPOS scores. The proportion of all patients with vital status and different dyspnea IPOS scores is shown in the y‐axis. IPOS, Integrated Palliative care Outcome Scale

Dyspnea IPOS scores decreased over 48 h (Figure 2). The percentage of patients with dyspnea also reduced, with 45%–52% of them having had no/mild dyspnea.

#### 3.2.5 Balanced outcomes between dyspnea intensity and communication capacity

Among 85 patients with dyspnea IPOS≥3 at the baseline, complete relief, partial relief, persistent dyspnea, being unable to communicate, and death were noted in 14 (17%), 36 (42%), 19 (22%), 5 (5.9%), and 11 (13%) patients, respectively, at 24 h. At 48 h, complete relief, partial relief, persistent dyspnea, being unable to communicate, and death were noted in 15 (18%), 32 (38%), 9 (11%), 10 (12%), and 19 (22%) patients, respectively.

### 3.3 Adverse events

Most adverse events were mild to moderate (CTCAE = 1–2), including nausea and delirium (Table 4).

## 4 DISCUSSION

### 4.1 Main findings

In this multicenter, prospective, cohort study, we found that a comprehensive pharmacological treatment algorithm is highly feasible, effective, and safe for terminal dyspnea in cancer patients.

### 4.2 What this study adds

The main significance of this study is the implementation of a visual flow chart of a comprehensive pharmacological treatment algorithm for terminal cancer dyspnea. Although international clinical guidelines suggest opioids and benzodiazepines for patients with terminal dyspnea, they do not show how to use them in clinical practice, leading to marked variability in practice even among palliative care specialists.^5^, ^10^, ^11^, ^16^, ^18^ In our recent proof‐of‐concept study, we indicated the feasibility of an opioid titration algorithm.^17^ The current study expanded the prior algorithm, by including comprehensive pharmacological treatment strategies. The implementation of this algorithm in clinical settings of primary palliative care would not only help oncologists and other non‐palliative care specialists provide quality care, but also help interdisciplinary professionals discuss goals of care with patients and families. In addition, the algorithm can be utilized for education purposes by visualizing otherwise invisible complex treatment strategies.

Notably, the multiple outcomes suggested the preliminary efficacy of the treatment algorithm. Patient‐reported outcomes are the gold standard, but not always available, as patients with terminal cancer and severe dyspnea often lose consciousness as death approaches and thereby cannot communicate.^8^, ^17^, ^21^ We thus included various outcomes including validated and novel measurements to maximize the interpretability.^2^, ^5^, ^8^, ^17^ As expected, patient‐rated NRS was available in only less than two‐thirds of patients at 48 h. To complement it, we conducted LOCF analysis, and used physician‐rated NRS and IPOS as well as respiratory rating; all of these consistently showed significant improvement in dyspnea (reduction of NRS greater than an MCID) or tachypnea. In addition, patient‐perceived goals were achieved and balanced outcomes were fulfilled in a majority of the patients. Moreover, adverse events were tolerable overall. To confirm its efficacy and safety, a randomized controlled trial is needed to compare the effects of the treatment algorithm and usual care is needed.

In contrast, approximately a third of participants did not experience dyspnea relief within 48 h. Similar findings were repeatedly reported by prior studies on terminal dyspnea.^5^, ^8^, ^17^ While the failure for improvement may reflect the progressive nature of terminal dyspnea toward death,^3^ it urges clinicians and researchers to challenge the status quo collaboratively. First, predictors of non‐responders to treatment should be identified. Second, the efficacy of more proactive treatment, such as rapid dose increment of opioids and prompt addition of low‐dose benzodiazepines as the second‐line treatment, should be investigated. The current treatment algorithm could be utilized as a control arm. Third, collaboration with basic scientists is warranted to better understand the underlying pathophysiology of terminal dyspnea and identify novel agents that could relieve dyspnea in a targeted fashion. Fourth, a holistic, interdisciplinary approach that integrates non‐pharmacological interventions should be developed and tested for patients with terminal dyspnea and their loved ones.^34^, ^35^, ^36^

### 4.3 Strengths and limitations

The study's strengths include patient enrollment at multiple sites, consecutive sampling, utilization of reliable and valid outcomes, and a novel effort to comprehensively visualize the pharmacological management by palliative care specialists, many of whom are involved in the development of national and international guidelines for cancer dyspnea.^11^, ^19^

However, several limitations should be noted. First, due to the observational nature and absence of a comparison arm, whether treatment with the algorithm is more effective than treatment without it could not be confirmed. Second, as the treatment was provided by palliative care specialists, the study findings might not be directly applied to general oncology settings. Thus, randomized controlled trials and well‐designed observational studies with comparison arms are required to confirm the effects of the treatment algorithm. Third, while our operational definition of goal achievement reflects clinical practice, it has not been validated. The use of palliative care physician determination that treatment goals were achieved in lieu of patient/family determination when patient could not communicate could be biased. Future research is needed to establish scales to measure the achievement of personalized symptom goals for patients with terminal dyspnea and impaired cognition. Fourth, several efficacy outcomes such as physician‐rated dyspnea NRS, IPOS, and physician‐rated improvement were assessed by treating physicians. While this reflects real‐world practice, there could potentially be a bias in reporting. Lastly, as the study was conducted only in Japan, the treatment algorithm may require future modification when used in other countries with different practice patterns.

## 5 CONCLUSION

In summary, the comprehensive pharmacological treatment algorithm is feasible, and may be effective and safe for cancer patients with terminal dyspnea. The algorithm could be easily instituted in other settings such as oncology and primary care globally. Its use may not only help clinicians improve care for such patients, but also help interdisciplinary professionals discuss goals of care with patients and families. Future studies are needed to demonstrate the effectiveness of the treatment algorithm in various primary palliative care settings.

## AUTHOR CONTRIBUTIONS

**Takashi Yamaguchi:** Conceptualization (equal); data curation (equal); investigation (equal); methodology (equal); project administration (equal); resources (equal); writing – review and editing (equal). **Kozue Suzuki:** Conceptualization (equal); data curation (equal); investigation (equal); methodology (equal); project administration (equal); resources (equal); writing – original draft (equal); writing – review and editing (equal). **Yoshinobu Matsuda:** Conceptualization (equal); data curation (equal); investigation (equal); methodology (equal); project administration (equal); resources (equal); writing – original draft (equal); writing – review and editing (equal). **Ryo Matsunuma:** Conceptualization (equal); data curation (equal); investigation (equal); methodology (equal); project administration (equal); resources (equal); writing – original draft (equal); writing – review and editing (equal). **Hiroaki Watanabe:** Conceptualization (equal); data curation (equal); investigation (equal); methodology (equal); project administration (equal); resources (equal); writing – original draft (equal); writing – review and editing (equal). **Tomoo Ikari:** Conceptualization (equal); data curation (equal); investigation (equal); methodology (equal); project administration (equal); resources (equal); writing – original draft (equal); writing – review and editing (equal). **Yoshihisa Matsumoto:** Conceptualization (equal); investigation (equal); methodology (equal); project administration (equal); writing – original draft (equal); writing – review and editing (equal). **Kengo Imai:** Conceptualization (equal); data curation (equal); investigation (equal); methodology (equal); project administration (equal); resources (equal); writing – original draft (equal); writing – review and editing (equal). **Naosuke Yokomichi:** Conceptualization (equal); data curation (equal); investigation (equal); methodology (equal); project administration (equal); resources (equal); writing – original draft (equal); writing – review and editing (equal). **Satoru Miwa:** Conceptualization (equal); data curation (equal); investigation (equal); methodology (equal); project administration (equal); resources (equal); writing – original draft (equal); writing – review and editing (equal). **Toshihiro Yamauchi:** Data curation (equal); investigation (equal); methodology (equal); project administration (equal); resources (equal); writing – original draft (equal); writing – review and editing (equal). **Soichiro Okamoto:** Data curation (equal); investigation (equal); methodology (equal); project administration (equal); resources (equal); writing – original draft (equal); writing – review and editing (equal). **Satoshi Inoue:** Data curation (equal); investigation (equal); methodology (equal); project administration (equal); resources (equal); writing – original draft (equal); writing – review and editing (equal). **Akira Inoue:** Conceptualization (equal); investigation (equal); methodology (equal); project administration (equal); resources (equal); writing – original draft (equal); writing – review and editing (equal). **Tatsuya Morita:** Conceptualization (equal); data curation (equal); investigation (equal); methodology (equal); project administration (equal); resources (equal); supervision (equal); writing – original draft (equal); writing – review and editing (equal). **Eriko Satomi:** Conceptualization (equal); funding acquisition (supporting); investigation (equal); methodology (equal); project administration (equal); resources (equal); supervision (equal); writing – original draft (equal); writing – review and editing (equal).

## FUNDING INFORMATION

This work was supported in part by Japan Hospice Palliative Care Foundation, Health, Labour, and Welfare Sciences Research Grant (19EA1011, 22EA1004), and JSPS KAKENHI Grant Number 20 K20618.

## CONFLICT OF INTEREST

No conflict of interest to declare.

## Data Availability

The data that support the findings of this study are available from the corresponding author, Masanori Mori, upon reasonable request. All authors agree to the journal to review the data if needed.
